# Mayer-Rokitansky-Kuster-Hauser syndrome complicated with giant mucinous cystadenoma and inguinal herniation: case report

**DOI:** 10.1093/omcr/omae036

**Published:** 2024-04-25

**Authors:** Artha Falentin Putri Susilo, Alfonsus Zeus Suryawan, Kevin Dominique Tjandraprawira, Anita Rachmawati

**Affiliations:** Department of Obstetrics and Gynecology, Universitas Padjadjaran, Bandung, West Java, Indonesia; Department of Obstetrics and Gynecology, Universitas Padjadjaran, Bandung, West Java, Indonesia; Department of Obstetrics and Gynecology, Universitas Padjadjaran, Bandung, West Java, Indonesia; Department of Obstetrics and Gynecology, Universitas Padjadjaran, Bandung, West Java, Indonesia

**Keywords:** MRKH syndrome, giant mucinous cystadenoma, mullerian system, inguinal hernia

## Abstract

Introduction: Coexistence of Mayer-Rokitansky-Kuster-Hauster syndrome (MRKH) with other conditions is rare, especially when MRKH was found in a young woman presenting with ovarian malignancy. This case report wishes to highlight MRKH complicated with giant mucinous cystadenoma and bilateral inguinal hernia. Case report: A 22-year-old nulligravid woman was admitted with primary amenorrhea and abdominal mass. Abdominal examination revealed a cystic mass 25 × 25 × 20 cm in size and a vagina 1 cm in length. Pelvic magnetic resonance imaging (MRI) showed a giant multiloculated left ovarian mass amidst the absence of uterus. During the surgery, the giant multiloculated cystic mass was identified as mucinous cystadenoma on frozen section. Bilateral medial inguinal hernia was also identified. Discussion: MRKH coexisting with other disease is rare but considering other structures arising from paramesonephric duct (PMD) may exist, allows the possibility of other structural anomalies. Conclusions: The present report illustrates a rare case of MRKH syndrome with giant ovarian cystadenoma and inguinal hernia.

## INTRODUCTION

Mayer-Rokitansky-Kuster-Hauser (MRKH) syndrome is congenital disorder which includes vaginal agenesis accompanied with variable mullerian ducts anomalies and sometime accompanied by renal, skeletal, or auditory abnormalities [1]. Coexistence with other pathologies is rare, let alone ovarian malignancy in a young female. According to the author’ knowledge, this was the first case in Indonesia and one of the very few cases in the world. This case report wishes to highlight MRKH complicated with giant mucinous cystadenoma and bilateral inguinal hernia.

## CASE REPORT

**Figure 1:**
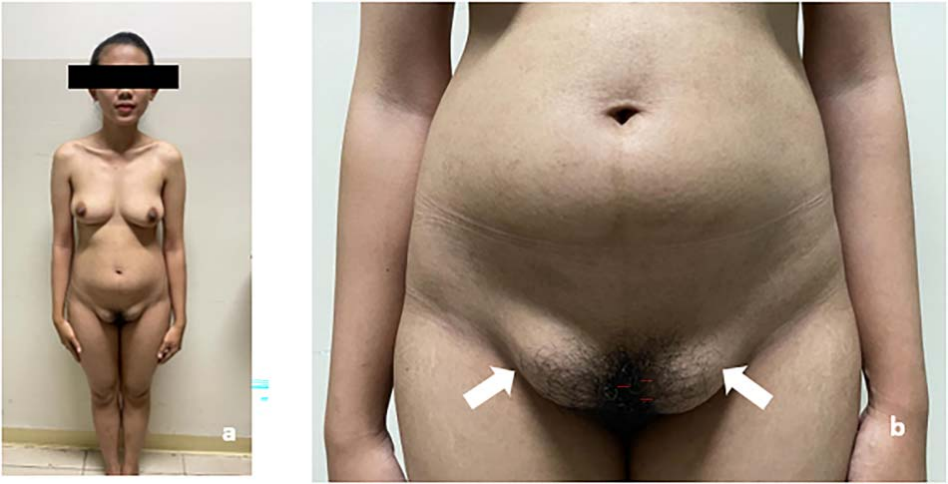
Patient physical stature. (**a**) Patient has tanner 4 for breast; and tanner 3 for pubic hair; (**b**) Abdominal mass and bilateral inguinal hernia was present (white arrow).

A nulligravid 22-year-old woman presented to our clinic complaining of primary amenorrhea and abdominal mass. She was concerned about her primary amenorrhea and had noted a sudden abdominal enlargement since 4 months before admission. The patient was Tanner stage 4 for pubic hair and Tanner stage 4 for her breasts (Figure 1). Physical examination revealed no hearing, cardiovascular, respiratory, and skeletal anomalies. Abdominal examination revealed a cystic mass measuring 25 × 25 × 20 cm. External genitalia and external urethral meatus were normal. Vagina was 1 cm in length. Bilateral inguinal hernia was present.

**Figure 2:**
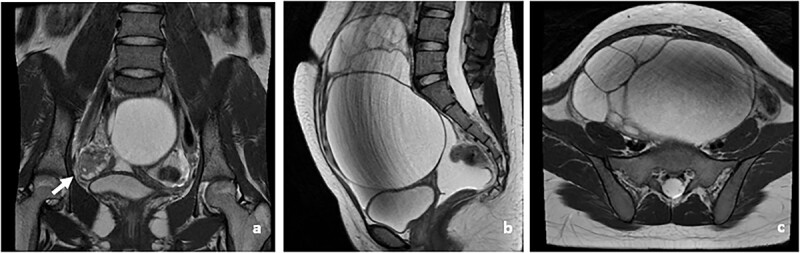
Patient MRI (**a**). Pelvic MRI showing normal right ovary (white arrow) (**b**). Sagital view of the left ovarian mass (**c**). Transverse view of the left ovarian mass.

**Figure 3:**
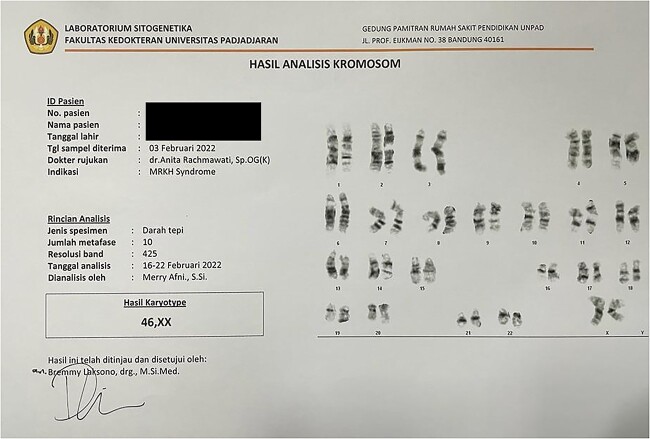
Patient Chromosome Analysis. Results shows patient karyotype was 46 XX.

Abdominal ultrasound revealed a multiloculated right ovarian cyst with clear borders, with intralesional vascularization; minimal ascites and notably, the absence of uterus. A confirmatory pelvic magnetic resonance imaging (MRI) showed a multiloculated left ovarian mass, 16,5 × 11 × 16,5 cm in size (suspected endometriosis or mucinous cystadenoma); intraperitoneal ascites with no lymphatic node enlargement; normal right ovary and no uterus (Figure 2). Her karyotype was 46, XX. Fluorescence in situ hybridization (FISH) analysis were not performed on this case (Figure 3).

The patient expressed a strong desire for her cystic mass to be removed through surgery. The patient expressed her plans on getting a future vaginoplasty should she wish to get married.

**Figure 4:**
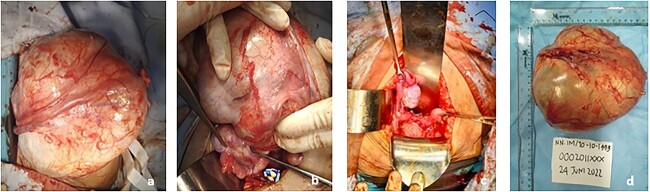
Intraoperative findings. (**a** and **b**) Mass gross appearance; (**c**) Right normal ovary and structural agenesis; d. mass after it was taking out with estimated size 20 × 18 × 15 cm.

A salpingo-oophorectomy was performed after the identification of a left ovarian multiloculated cystic mass measuring 20 × 18 × 15 cm (Figure 4). Her frozen section indicated a mucinous cystadenoma. A bilateral herniorrhaphy was performed subsequently. The bilateral medial inguinal hernia was found to only contain peritoneal fluid with no discovery of omentum, digestive tract nor ovaries.

Pathology anatomy analysis reveals the mass is a mucinous cystadenoma consistent with the frozen section result. Molecular markers like KRAS/BRAF mutations were not examined due to limitation of our national healthcare system.

## DISCUSSION

Mayer-Rokitansky-Kuster-Hauser (MRKH) syndrome is congenital disorder which includes vaginal agenesis accompanied with variable Mullerian ducts anomalies and sometime accompanied by renal, skeletal, or auditory abnormalities [1]. MRKH is divided into two categories, type I is isolated MRKH and type II is associated with extragenital abnormality (renal, skeletal, and others) [2]. The cause of this anomaly could be traced back to embryogenesis as a disruption in morphogenesis of fetal genito-reproductive tract. This syndrome is caused by the failure of the paramesonephric duct (PMD) to form a uterus, either due to or agenesis [2, 3]. The ovaries and endocrine status are normal and functional in MRKH syndrome. Karyotyping is essential for MRKH and our patient is 46, XX with no mosaicism [4].

The coexistence of MRKH with other diseases is rare but has been reported elsewhere. Ovarian masses have been reported, very rarely, to coexist and they may be benign or malignant [5]. Among benign ovarian tumors, mucinous cystadenomas account for 10%–15% of all cases [6]. Benign mucinous cystadenomas make up the majority (81%) of mucinous tumors. This type of tumor if left untreated could grow exponentially big [7]. Pathogenesis of ovarian tumor is still unclear, but it’s hypothesized to arise from uninterrupted ovulatory cycle which leads to mutations and tumor formation. Mucinous ovarian tumors are usually linked with KRAS mutation. KRAS mutations do occur in benign and particularly in malignant mucinous ovarian tumors, suggesting early mutation [8, 9]. Other than ovary origins, it’s been suggested Müllerian cortical inclusion cysts, presumably could undergo proliferation and differentiation. This leads to formation of ovarian tumor [10]. However, it is important to note that the pathogenesis is still unclear and the current literature can only offer theoretical suggestions thus far.

The gold standard of treatment of any suspected ovarian mass includes intact removal of the involved adnexa with intraoperative pathological evaluation, and operative procedure following the pathologic findings [11]. Salpingo-oophorectomy was performed in our patient and the frozen section and the subsequent pathology report confirmed the finding of mucinous cystadenoma.

The origin of mucinous cystadenoma in this case may be attributed to two sources. The first possibility is its arising from the remnants of the Mullerian system. This is the most favored theory, but it is inconsistent. Thus, it is possible that the ovarian mass arose from the secondary Mullerian system [12]. Secondary Müllerian system itself are designate structures lined by Müllerian epithelium found outside the uterus, cervix, and fallopian tubes [13]. Lancet *et al* stated this system represent embryological remnants of the proximal Müllerian ducts which located within the ovarian hilum [12]. Inclusion of this system leads formation of ovarian tumor through proliferation and differentiation of the cells [10, 12].

Our finding of bilateral inguinal hernia is even rarer. The possibility of ovarian herniation was considered. In 1984 Thompson offered the hypermobility hypothesis in which the failure of fusion of Mullerian ducts could lead to herniation of ovary and other adnexae into the inguinal canal [14]. In 2012, Okada reported a similar case whilst suggesting the elongation of ovary suspensory ligament due to other structures abscence could be the cause of the herniation into the inguinal canal [15]. in our case, pelvic MRI ruled out this possibility, further confirmed by intraoperative finding of a peritoneal fluid pouch as the hernia. However, the displacement of adnexal structures due to high intra-abdominal pressures should not be ruled out before confirmatory imaging such as MRI as it is still theoretically possible [16].

There remains little information on MRKH and ovarian malignancy. Including our report, only 7 cases have been reported worldwide of MRKH complicated with an ovarian mass. Our report is the first in Indonesia [16]. Our report should highlight that despite its rarity and lack of clear pathophysiology, ovarian mass complicating MRKH is possible.

## CONCLUSION

A giant ovarian cystadenoma coexisting with bilateral inguinal hernia and MRKH is exceedingly rare. No unifying pathogenesis is yet available to explain such coexistence. Timely diagnosis and management are paramount.

## TAKE-HOME MESSAGE

1. Association of MRKH with ovarian anomalies such as tumor and/or malignancy is possible and should be taken into consideration.
2. Ovarian hernia should not be ruled out until it was proven to preserve fertility.
3. MRI is helpful to identify mass and anatomical structure in patients with MRKH and ovarian mass.

## Data Availability

Not applicable.

## REGISTRATION OF RESEARCH STUDIES

Registration of research is not applicable in our case.
